# Synergistic activity and molecular modelling of fosfomycin combinations with some antibiotics against multidrug resistant *Helicobacter pylori*

**DOI:** 10.1007/s11274-022-03289-2

**Published:** 2022-04-29

**Authors:** Ahmed Megahed Abouwarda, Tarek Abdelmonem Ismail, Wael Mohamed Abu El-Wafa, Ahmed Hassan Ibrahim Faraag

**Affiliations:** 1 Department of Microbiology, General Division of Basic Medical Sciences, Egyptian Drug Authority (EDA), Formerly National Organization for Drug Control and Research (NODCAR), Giza, Egypt; 2 Faculty of Science, Botany and Microbiology Department, Helwan University, Ain Helwan, Cairo, 11795 Egypt

**Keywords:** *Helicobacter pylori*, Fosfomycin, Metronidazole, Clarithromycin, Resistant, Synergism, Docking

## Abstract

Antibiotic resistance represents the main challenge of *Helicobacter pylori* infection worldwide. This study investigates the potential bactericidal effects of fosfomycin combinations with clarithromycin, metronidazole, ciprofloxacin, amoxicillin, rifampicin, and doxycycline against thirty-six *H. pylori* strains using the checkerboard and time-kill assay methods. The results showed that ≥ 50% of the strains were resistant to the six antibiotics. Remarkably, only six strains exerted resistance to these antibiotics, with the minimum inhibitory concentrations (MICs) ranges of (3.2–12.8 mg/l), (32–256 mg/l), (3.2–51.2 mg/l), (3.2–25.6 mg/l), (1.6–3.2 mg/l), and (25.6 > 51.2 mg/l), respectively. The seven antibiotics were evaluated through in silico studies for their permeability and ability to bind UDP-N-acetylglucosamine1-carboxyvinyltransferase (MurA) of *H. pylori.* The results indicated that fosfomycin exhibited the highest predicted membrane permeability (membrane ∆G insert = − 37.54 kcal/mol) and binding affinity (docking score = − 5.310 kcal/mol) for *H. pylori* MurA*,* compared to other tested antibiotics. The combinations of fosfomycin with these antibiotics exerted synergistic interactions (Fractional inhibitory concentration, FIC index < 1) against the six strains. Importantly, the combinations of fosfomycin with clarithromycin, doxycycline and rifampicin achieved bactericidal effects (reduction ≥ 3.0 Log_10_ cfu/ml) against the most resistant *H*. *pylori* strain. Notably, these effects increased with presence of metronidazole, which enhanced the activity of the fosfomycin combination with amoxicillin from a weak inhibition to bactericidal effect. This study provides evidence that the combination of fosfomycin with either clarithromycin, amoxicillin, doxycycline, or rifampicin (especially with the presence of metronidazole) could be a promising option for treating MDR *H*. *pylori* infection.

## Introduction

*Helicobacter pylori* is a Gram negative, microaerophilic, motile, and spiral-shaped bacterium. It represents one of the most frequent bacterial human infections worldwide (Lien et al. 2019). This clinically-important bacterium is linked to many gastrointestinal diseases, including gastritis, peptic ulcer, gastric adenocarcinoma, and mucosa-associated lymphoid tissue lymphoma. Additionally, there were extra-gastric diseases associated with *H. pylori* infection, including cardiovascular, respiratory, extra-gastroduodenal digestive, neurological, dermatological, autoimmune and growth disorders (Flores-Treviño et al. 2018; Gravina et al. 2018).

There are more than 4.4 billion patients worldwide estimated to have *H. pylori* infection (Hooi et al. 2017), which significantly influenced by age, sex, geographical regions, ethnicity, and socio-economic factors (Reffert and Smith 2014; Hooi et al. 2017; Zamani et al. 2018; Hu et al. 2020). Meanwhile**,** Africa has the highest rate of *H. pylori* infection worldwide, followed by South America and Western Asia, with prevalence of 70.1, 69.4% and 66.6%, respectively (Reffert and Smith 2014). In Egypt, the prevalence of *H. pylori* infection ranged from 60 to 90% (Mohamed et al. 2014; El-Khlousy et al. 2016; Ismail and Mostafa 2018).

Currently, antibiotic resistance is the main challenge in the management of *H. pylori* infection worldwide. The recent systematic reviews and meta-analyses demonstrated that the primary and secondary resistance rates to clarithromycin (CLA), metronidazole (MET) and ciprofloxacin (CIP) exceeded 15% (alarming levels) in all developed and developing countries (Safavi et al. 2016; Gong and Yuan 2018; Savoldi et al. 2018; Lien et al. 2019; Hu et al. 2020). It is noteworthy to mention that Africa had the second rates of amoxicillin (AM) and doxycycline (DO) resistance in *H. pylori* infection worldwide, with a prevalence rate of 65.5 and 43.9%, respectively(Arslan et al. 2017). Despite, the global rifampicin (RIF) resistance in *H. pylori* is limited, the infection with rifampicin-resistant *H. pylori* has significantly increased in some geographical regions; America, Europe and Oceania, with a prevalence rate of 46.1, 33.3 and 23.1%, respectively (Flores-Treviño et al. 2018). Thus, alternative safe and effective treatment regimens for resistant *H. pylori* infection are urgently needed.

Fosfomycin (FOS) is a broad-spectrum antibiotic, with putative activity against multidrug resistant (MDR) Gram-positive and Gram-negative pathogens. It inhibits the early stage of the bacterial cell wall synthesis. Several studies have investigated the synergistic effects of FOS when combined with other antibiotics that act via a different mechanism of action, thereby allowing for decreased dosages and lower toxicity (Zhanel et al., 2018; Davis et al., 2020; Abu El-Wafa and Ibrahim, 2020; Seok et al., 2020). In this study we investigate the potential bactericidal effects of FOS combinations with antibiotics (CLA, CIP, AM, DO and RIF) against MRD *H. pylori* in the presence and absence of MET.

## Materials and methods

### Bacterial strains and growth conditions

The *H. pylori* strains (*n* = 36) used in this study were previously isolated from gastric biopsy specimens from patients with gastric and peptic ulcer. The strains were previously identified based on standard biochemical and molecular (16S rRNA) approaches (Mostafa et al. 2018). The *H. pylori* cultures were separately preserved with 50% (v/v) glycerol at − 70 °C until use. All the experiments of this study were carried out under microaerophilic conditions using an anaerobe jar (Oxoid, Ltd) with microaerophilic gas-generating kit (code no. BR 56; Oxoid, Ltd).

### Antibiotic susceptibility testing

The susceptibility of thirty six *H. pylori* strains to FOS, CLA, MET, CIP, AM, RIF and DO (European pharmacopoeia reference standards) was evaluated through the determination of MIC using agar plate dilution method according to European Committee on Antimicrobial Susceptibility Testing guidelines (EUCAST, 2020). Briefly, twofold serial dilutions of these antibiotics were separately performed in Mueller–Hinton agar (MHA, Oxoid) plates supplemented with 5% defibrinated sheep blood. Each agar plate was inoculated with 2 μl /spot of each *H. pylori* inoculum (1 × 10^6^ cfu/ml). The final concentrations of FOS and MET were ranging from 0.5 to 512 mg/l, whereas the final concentrations of CLA, CIP, AM, RIF and DO were ranging from 0.00315 to 51.2 mg/l. Following the inoculation, the plates were dried at room temperature and then incubated at 37 ℃ for 4 days. The MIC was defined as the lowest concentration that inhibited visible growth of bacteria.

### Homology modelling

The homology model of UDP-N-acetylglucosamine 1-carboxyvinyltransferase (MurA) sequence was performed using the SWISS-MODEL prediction tools (https://swissmodel.expasy.org/). A template search with BLAST and HHblits has been performed against the SWISS-MODEL template library based on the ProMod3 target template alignment and evaluated using the QMEAN score function (Guex et al. 2009; Benkert et al. 2011; Remmert et al. 2012; Bienert et al. 2017; Bertoni et al. 2017; Waterhouse et al. 2018) The quaternary structure and InterPro protein families and domains tool analysis of MurA of the target *H. pylori* sequence (UniProtKB-ID: P56189) were used to build the three dimensional (3D) model with template sequence of high sequence identity according to (Kessler et al. 1982; Bertoni et al. 2017; Blum et al. 2021).

### *In Silico* Docking Study

**Fig. 1:**
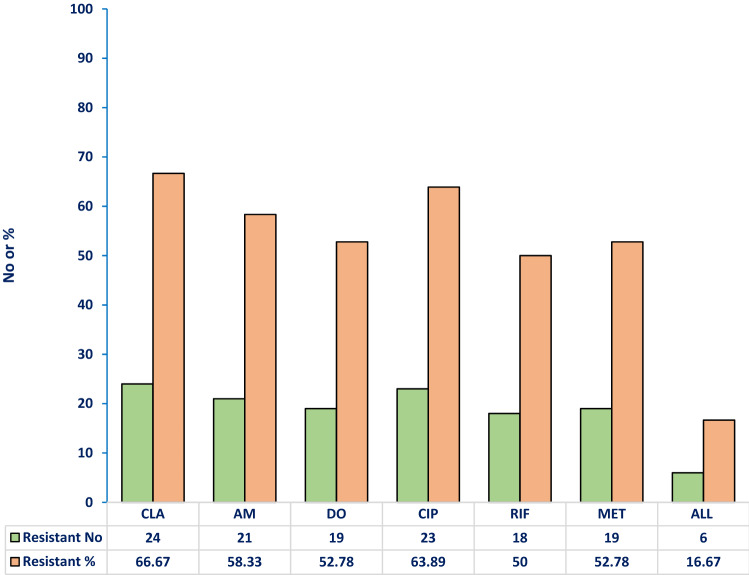
Susceptibility of thirty-six *Helicobacter pylori* strains to six different antimicrobial agents. No number, %, percentage, CLA clarithromycin, AM amoxicillin, DO doxycycline, CIP ciprofloxacin, RIF rifampicin, MET metronidazole

The docking experiment was performed with Glide’s Extra Precision (XP) program from Schrödinger 16.4 (Friesner et al. 2006). The analysis was conducted using the following ligands: FOS, CLA, MET, CIP, AM, RIF and DO which were retrieved from PubChem Bioassay. Maestro 11.9 and LigPrep 2.4 applications have been used for the preparation of the ligand. For the docking analysis, MurA homology model of the crystallographic structure was used. Figure 1 revealed the 3D structure of MurA The grid size was defined as 20 Å by default for each protein. The MacroModel of Schrödinger software was used to reduce energy for all ligands (Jorgensen et al. 1996; Kaminski et al. 2001; Schrodinger 2013).

The pKa value of each ionizable atom in FOS was determined by utilizing the empirical pKa panel of Schrodinger software. Additionally, the generation of the most likely ionized and tautomerized states of FOS in different pH levels ranging from 3 to 11 was also evaluated (Balogh et al. 2012; Schrodinger 2013).

### *In silico* models for predicting membrane permeability

Computationally, membrane permeability prediction of FOS, CLA, MET, CIP, AM, RIF and DO using the membrane permeability prediction tools in the physics-based permeability prediction module within the Schrödinger’s Small-Molecule Drug Discovery Suite 12.8 based on Membrane ∆G Insert Eq. (1) (Rezai et al. 2006; Leung et al. 2016; Schrödinger Release 2019–1 2019). Energy minimization for all ligands was performed using the macro-Model of Schrödinger’s software (Jorgensen et al. 1996; Kaminski et al. 2001; Schrodinger 2013).

1 $$Membrane \, \Delta G\,Insert\,\, = \,\,energy \, of\,Membrane\,HDLD\, + \,Membrane\,State\,Penalty$$

### Determination of fractional inhibitory concentrations

The FIC of double combinations of FOS with CLA, CIP, AM, RIF, DO and MET against *H. pylori* strains was determined by checkerboard microdilution method (Kim et al. 2016). Briefly, FOS was serially diluted twofold in a horizontal orientation, whereas CLA, CIP, AM, RIF, DO and MET were serially diluted twofold in a vertical orientation. The final concentrations of FOS or MET in 200 μl Mueller–Hinton broth (MHB) were ranging from 512 to 1.0 mg/l, whereas the final concentrations of CLA, CIP, AM, RIF and DO in 200 μl MHB were ranging from 5.12 to 0.00315 mg/l. The inoculum size of each test strain was approximately 1 × 10^6^ cfu/ml. Inoculated and un-inoculated wells (containing 200 μl MHB) were considered as positive and negative controls, respectively. Following the inoculation, the plates were incubated for 48 h at 37 °C. Additionally, FICs of triple combinations of FOS/MET with CLA, CIP, AM, RIF and DO were determined by the same above-mentioned method. FIC index of the combinations was calculated by the sum of the FIC of each antibiotic alone (MIC of antibiotic in combination/MIC of antibiotic alone). FIC index of antibiotic combinations defined as synergy (ΣFIC ≤ 1), indifference (1.0 < ∑FIC ≤ 4) or antagonism (ΣFIC > 4) (Kamatou et al. 2006). The MICs of the synergistic antibiotic combinations were further tested against the representative strain by a time-kill assay.

### *In vitro* time kill assays

The bactericidal activities of antibiotics (MET, CLA, CIP, AM, RIF and DO) and their respective combinations with FOS (in the presence/absence of MET) against the representative strain were evaluated by performing time-kill assay (Coudron and Stratton 1995). Briefly**,** single antibiotics, double and triple antibiotic combinations were performed in sterile MHB, and then inoculated with 10 µl of 48 h culture of test strain. The final inoculum size of each test strain in 50 ml MHB was 1 × 10^6^ cfu/ml. Aliquots were taken at different time intervals (0, 3, 6 and 24 h) and serial tenfold dilutions were prepared in sterile sodium chloride solution (0.9%, w/v) as needed. Three replicates of each diluent were spotted on MHA supplemented with 5% defibrinated sheep blood, dried at room temperature and then incubated 4 days at 37 °C (Inoculated and un-inoculated MHB were considered as positive and negative controls). The data were analyzed by using mean colony counts (Log_10_ cfu/ml) from the replicates of each diluent at each time interval. The limit of quantification was 2 Log_10_ cfu/ml. The synergy of the combination was defined as a 2 Log_10_ cfu/ml decrease compared with the most active antibiotic in this combination, whereas the bacteriostatic and bactericidal effects were defined as 2 and 3 Log_10_ cfu/ml decease relative to the initial inoculum, respectively.

## Results

The susceptibility of 36 *H. pylori* strains to six different antimicrobial agents was estimated through the determination of the minimum inhibitory concertation (MIC) using the agar plate dilution method. As shown in Fig. 1, 24 (66.67%) strains were resistant to CLA, 23 (63.89%) strains were resistant to CIP, 21 (58.33%) strains were resistant to AM, 19 (52.78%) strains were resistant to DO and MET, and 18 (50%) strains were resistant to RIF. Remarkably, six *H. pylori* strains were found resistant to the six tested antibiotics.

The MICs of the seven different antibiotics against the six MDR *H. pylori* strains are summarized in Table 1. The results showed that all strains were resistant to CLA, MET, CIP, AM, RIF and DO. No interpretive criteria are provided for FOS on *H. pylori* in either the CLSI or the EUCAST. The MICs ranges of the six antibiotics against the test strains were (3.2–12.8 mg/l), (32–256 mg/l), (3.2–51.2 mg/l), (3.2–25.6 mg/l), (1.6–3.2 mg/l), and (25.6–> 51.2 mg/l), respectively. The MIC of FOS against these strains was ranging from 128 to 256 mg/l. Additionally, all the test strains were classified as MDR since they exhibited resistance to more than two antibiotics related to different antibiotic categories. Notably, *H. pylori* HP-1 exhibited the highest MIC values of all tested antibiotics. Thus, this strain was selected as a representative strain for time kill studies.

**Table 1:** The minimum inhibitory concentrations of the seven different antibiotics against the most resistant *H. pylori* strains

Strains	MIC of antibiotics (mg/l)/ Susceptibility pattern*							Resistance pattern
	CLA	AM	DO	CIP	RIF	MET	FOS	
HP-1	12.8/R	25.6/R	> 51.2/R	51.2/R	3.2/R	256/R	256/NL	MDR
HP-2	3.2/R	6.4/R	> 51.2/R	3.2/R	1.6 /R	256/R	128/NL	MDR
HP-3	12.8/R	3.2/R	> 51.2/R	3.2/R	3.2/R	64/R	256/NL	MDR
HP-4	6.4/R	25.6/R	25.6/R	25.6/R	3.2/R	128/R	128/NL	MDR
HP-5	3.2/R	12.8/R	25.6/R	25.6/R	3.2/R	128/R	128/NL	MDR
HP-6	3.2/R	3.2/R	> 51.2/R	3.2/R	3.2/R	32/R	128/NL	MDR

^*^According to EUCAST, *MIC* minimum inhibitory concentration, *R* resistant, *NL* not listed in EUCAST guideline, *MDR* multidrug resistant, *FOS* fosfomycin, *MET* metronidazole, *CLA* clarithromycin, *CIP* ciprofloxacin, *AM* amoxicillin, *DO* doxycycline, *RIF* rifampicin

**Fig. 2:**
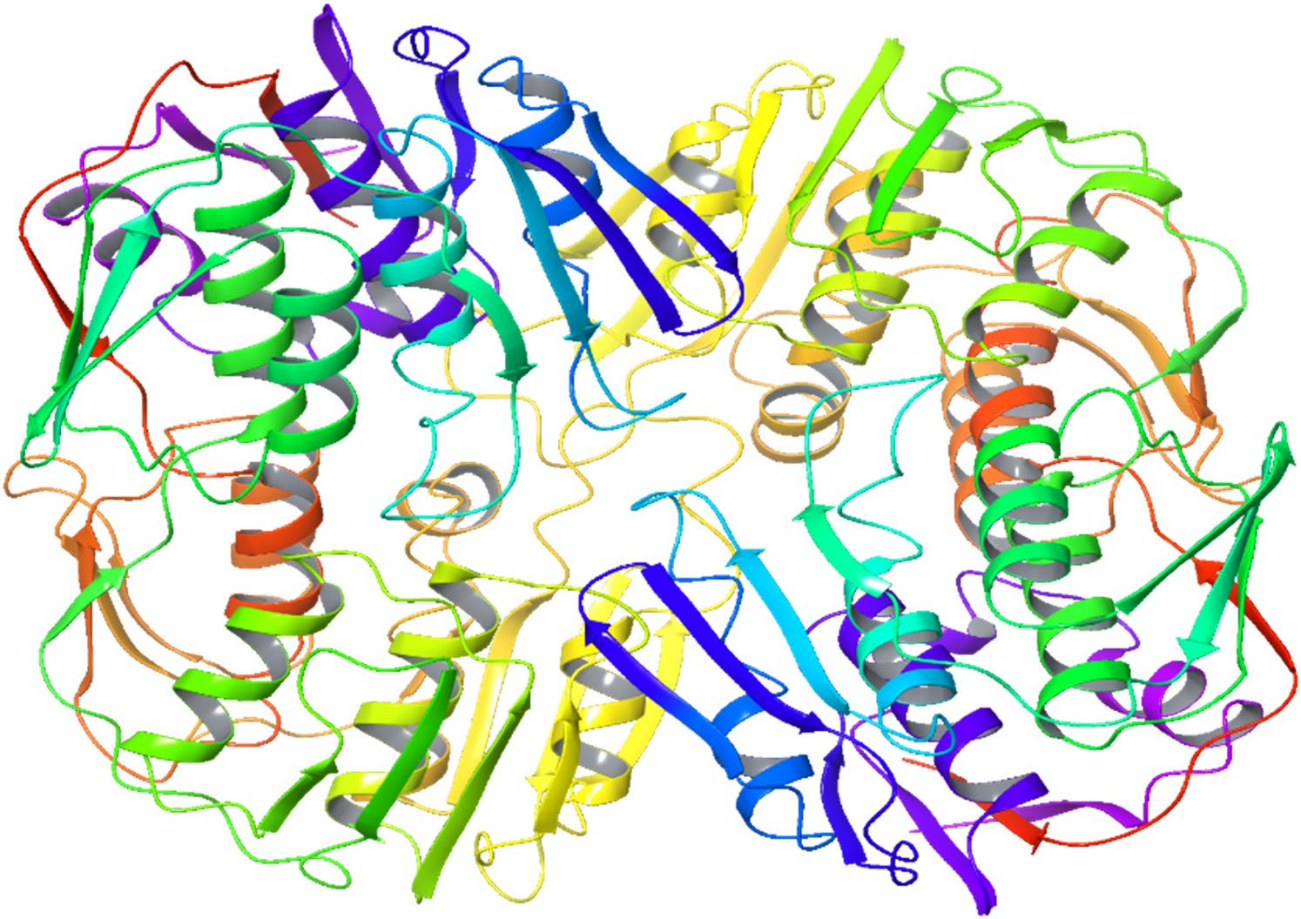
The 3D homology model of *Helicobacter pylori* MurA (UDP-N-acetylglucosamine enolpyruvyl transferase)

**Fig. 3:**
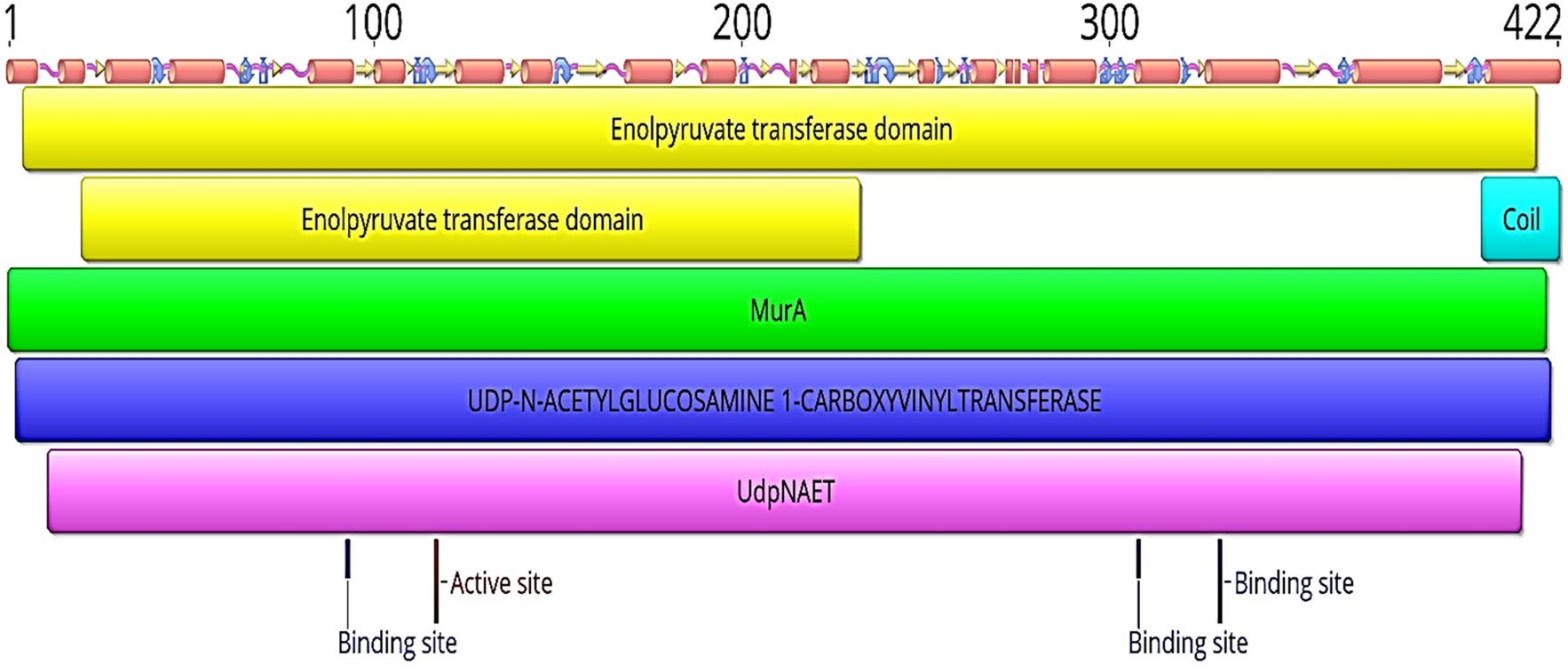
InterPro protein families and domains of *Helicobacter pylori* MurA

The 3D structure of *H. pylori* MurA was determined based on the principle of homology modeling, using of a templet (PDB:5UJS) from *Campylobacter jejuni* ATCC 700,819. The structure analysis indicated that *H. pylori* MurA showed 60.05% sequence identity with crystal structure of MurA protein from *Campylobacter jejuni* ATCC 700,819. Additionally, the InterPro protein families and domains of *H. pylori* MurA demonstrated that the Mur A possesses one active site contain Cys^117^ and 3 binding sites contain Arg^93^, Asp^308^ and Leu^330^, respectively (Figs. 2, 3).

**Fig.4:**
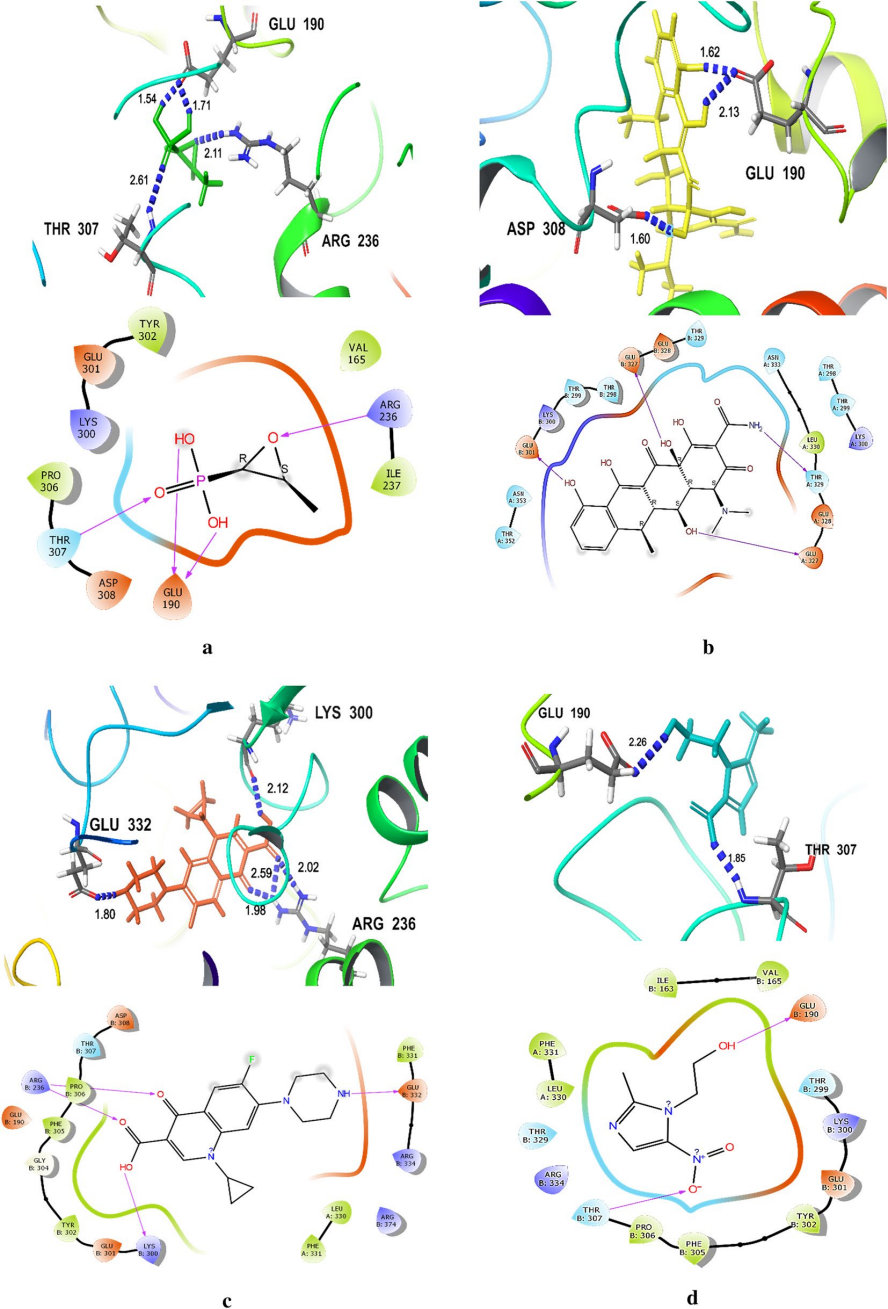

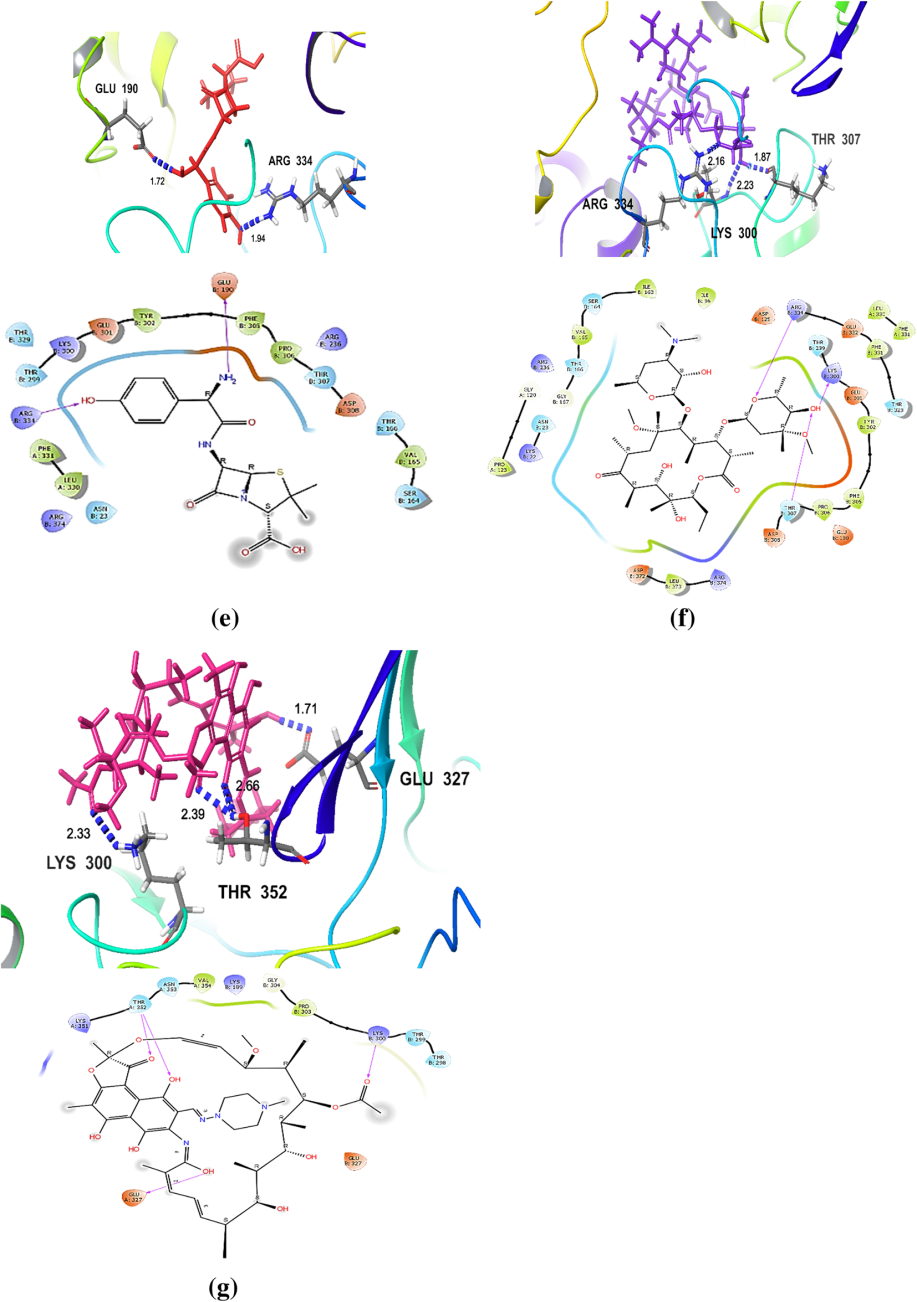
**a** The interaction between *Helicobacter pylori* Mur A and fosfomycin. **b** The interaction between *Helicobacter pylori* Mur A and doxycycline. **c** The interaction between *Helicobacter pylori* Mur A and ciprofloxacin. **d** The interaction between *Helicobacter pylori* Mur A and metronidazole. **e** The interaction between *Helicobacter pylori* Mur A and amoxicillin. **f** The interaction between *Helicobacter pylori* Mur A and clarithromycin. **g** The interaction between *Helicobacter pylori* Mur A and rifampicin. Arg arginine, Glu glutamic acid, Lys lysine, Thr threonine, PHE Phenylalanine, PRO Proline, ARG Arginine, ASP Aspartate, VAL Valine, SER Serine, TYR Tyrosine, LEU Leucine, ASN Asparagine, GLY Glycine, LEU Leucine, ILE Isoleucine, the blue dashed lines represent H-bonds and the numbers denote the distance of the H-bonds

The molecular docking study of the seven tested antibiotics to *H. pylori* MurA indicated that FOS exhibited the highest binding affinity with protein active site of *H. pylori* MurA, followed by DO, with docking scores equal to -5.310 and− 5.135 kcal/mol, respectively. Whereas, CIP, MET, and AM showed moderate binding affinity, with docking scores equal to − 4.744, − 4.549, and − 4.356 kcal/mol, respectively. The lower binding affinity with protein active site of *H. pylori* MurA was observed with CLA and RIF, with docking scores equal to − 3.887, and − 3.834 kcal/mol, respectively (Table 2 and Fig. 4a–g).

**Table 2:** The Docking scores and hydrogen bonds between legends and *H. pylori MurA*

Ligands	Free energy of binding (Kcal/mol)	Residues involved in hydrogen bonding	H-bonds distance (Å)	Number of hydrogen bonds
FOS	− 5.31	*Glu*^*190*^* (B)*, Arg^236^ (B), Thr^307^ (B)	1.54, 1.71, 2.11, 2.61	4H bonds
DO	− 5.14	*Glu*^*190*^* (B)*, Asp^308^ (B)	1.62, 2.13, 1.60	3H bonds
CIP	− 4.74	**Arg**^**236**^** (B)**, Lys^300^ (B), Glu^332^ (B)	1.98,2.02, 2.52, 2.12, 1.80	5H bonds
MET	− 4.55	Glu^190^ (B), Thr^307^ (B)	2.26, 1.85	2H bonds
AM	− 4.36	Glu^190^ (B), Arg^334^ (B)	1.72, 1.94	2H bonds
CLA	− 3.89	Lys^300^ (B), Thr^300^ (B), Arg^334^ (B)	2.23, 1.87, 2.16	3H bonds
RIF	− 3.83	Lys^300^ (B), Glu^327^ (B),* Thr*^*352*^* (A)*	2.33, 1.71, 2.39, 2.66	4H bonds

Amino acids form 2 & 3 hydrogen bonds are highlighted in italic & bold, respectively

*FOS* fosfomycin, *MET* metronidazole, *CLA* clarithromycin, *CIP* ciprofloxacin, *AM* amoxicillin, *DO* doxycycline, *RIF* rifampicin; *Arg* arginine, *Asp* aspartic, *Glu* glutamic acid, *Lys* lysine, *Thr* threonine

(A, B) protein chains A & B, respectively

**Fig. 5:**
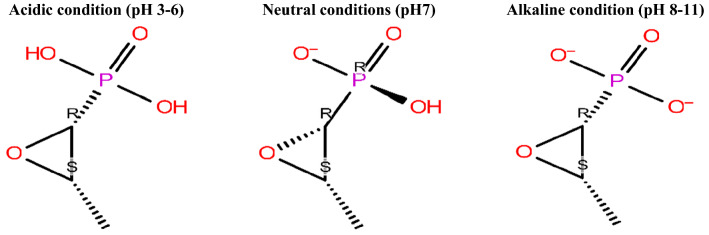
Effect of different pH levels on the total charges of FOS

**Fig. 6:**
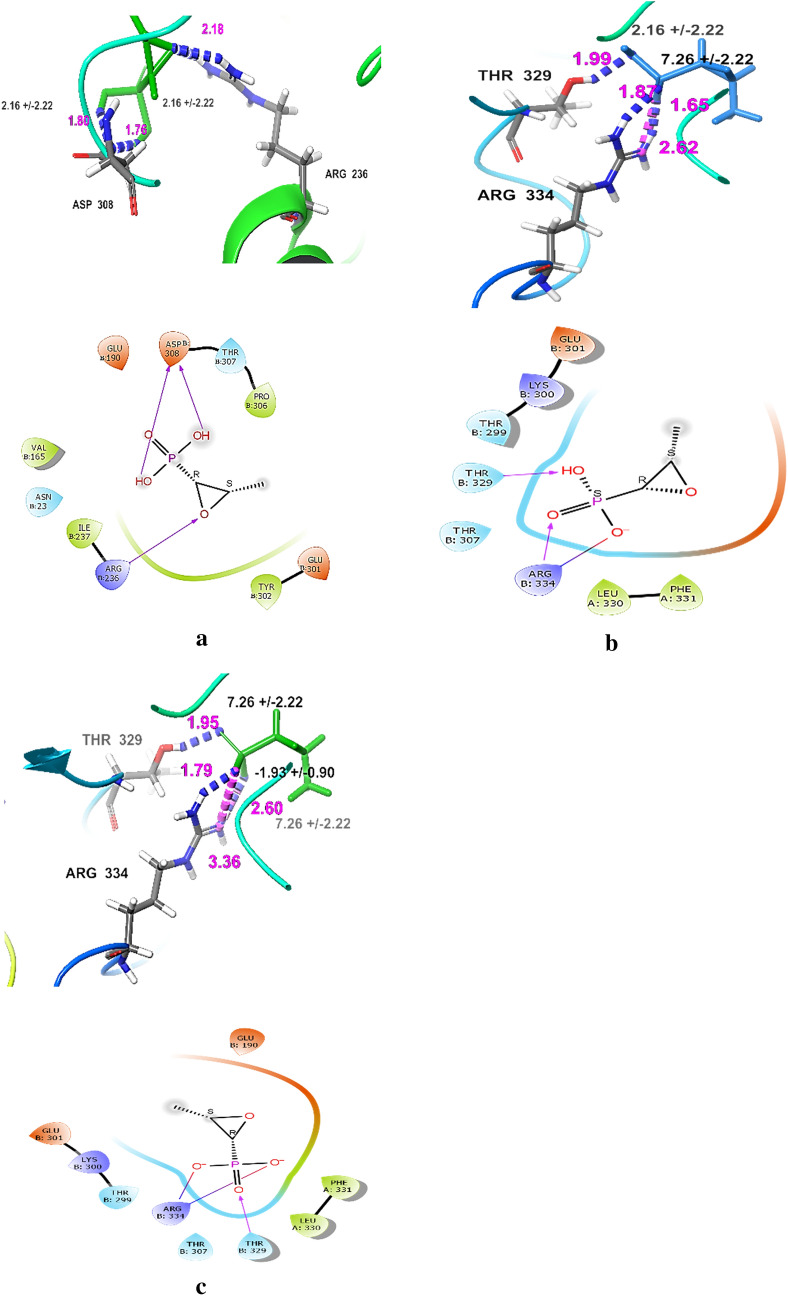
**a** The interaction between *Helicobacter pylori* Mur A and FOS under acidic condition (pH 3–6). **b** The interaction between *Helicobacter pylori* Mur A and FOS under neutral condition (pH 7). **a**, **c** The interaction between *Helicobacter pylori* Mur A and FOS under alkaline condition (pH 8–11). Arg arginine, Glu glutamic acid, Lys lysine, Thr threonine, PHE Phenylalanine, PRO Proline, ARG Arginine, ASP Aspartate, VAL Valine, SER Serine, TYR Tyrosine, LEU Leucine, ASN Asparagine, GLY Glycine, LEU Leucine, ILE Isoleucine, the blue dashed lines represent H-bonds and the numbers denote the distance of the H-bonds

The hydrogen (H) bonding interactions in the best docking are also described in Table 2 and Fig. 4a–g. The results showed that the maximum total number of the hydrogen (H) bonds between tested antibiotics and the protein active site of *H. pylori* Mur A was observed with CIP, which forms 5 H-bonds; 3H bonds with Arg^236^ and 2H bonds with Lys^300^ and Glu^332^, with bond lengths of 1.98, 2.02, 2.52, 2.12 and 1.80 Å, respectively, followed by FOS and RIF, which form 4H bonds; FOS forms 2H bonds with Glu^190^, and 2H bonds with Arg^236^ and Thr^307^, with bond lengths of 1.54, 1.71, 2.11 and 2.61 Å, respectively, REF forms 2H bonds with Thr^352^ and 2H bonds with Lys^300^ and Glu^327^, with bond lengths of 2.33, 1.71, 2.39 and 2.66 Å, respectively. Whereas, DO and CLA form 3H bonds; DO forms 2H bonds with Glu^190^ and H bond Asp^308^, with bond lengths of 1.62, 2.13 and 1.60 Å, respectively, CLA forms 3H bonds with Lys^300^, Thr^300^ and Arg^334^, with bond lengths of 2.23, 1.87 and 2.16 Å, respectively. The lower number of H-bonds between tested antibiotics and the protein active site of *H. pylori* Mur A was observed with MET and AM, which forms 2 H-bonds; MET forms 2H bond with Glu^190^ and Thr^307^, with bond lengths of 2.26 and 1.85 Å, respectively, AM forms 2H bonds with Glu^190^ and Arg^334^, with bond lengths of 1.72 and 1.94 Å, respectively,

The effect of different pH levels on the docking score of FOS are summarized in Table 3 and Figs. 5, 6a-c. The obtained results revealed that the docking score of FOS was increased under alkaline conditions, reaching − 7.456 kcal/mol in the pH values range of 8–11 and possessing two negative charges with the formation of 3H bonds with Arg^234^ and Thr^329^. Under neutral condition (pH 7), the docking score of FOS was − 5.708 kcal/mol, possessing one negative charge and forming 3H bonds with Arg^234^ and Thr^329^. Oppositely, under acidic conditions, the docking score of FOS deceased to 2.945 kcal/mol in the pH values ranging from 6 to 3, and it was found in the 0 state with very low binding affinity, forming 3H bonds with Arg^234^ and Asp^308^.

**Table 3:** Effect of different pH levels on the docking score and state penalty of FOS and its interaction with *H. pylori* MurA

pH value	State Penalty (kcal/mol) *	Total charge (Tot Q)	Docking score (kcal/mol)	Interacting amino acids	Number of hydrogen bonds	H-bonds distance (Å)	Salt bridge (Å)
3–6	6.5975	0	2.945	Arg ^234^ (B), Asp ^308^ (B)	3 H bonds	1.76, 1.80, 2.18	0
7	0.2534	− 1	− 5.708	Arg ^234^ (B), Thr ^329^ (B)	3 H bonds	1.65, 1.87, 1.99	2.62
8–11	0.6255	− 2	− 7.456	Arg ^234^ (B), Thr ^329^ (B)	3 H bonds	1.79n, 1.95, 2.60	2.60, 3.36

State Penalty is free energy of this state in the ensemble of states generated, in kcal/mol, Tot Q is total charge in this state

*Arg* arginine, *Asp* aspartic, *Thr* threonine

(B) protein chain B

**Fig. 7:**
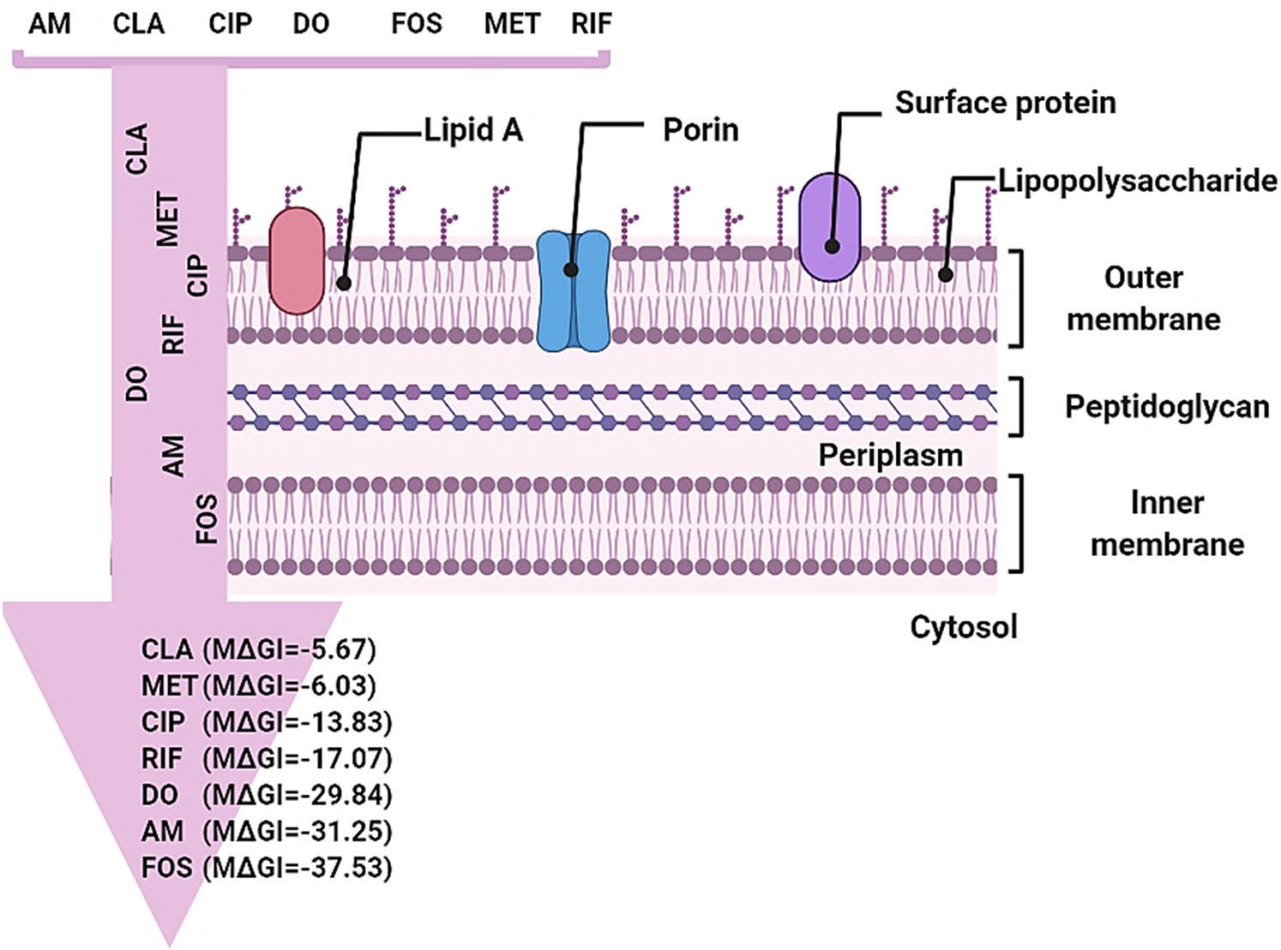
Membrane permeability of seven different antibiotics in *Helicobacter pylori*. FOS fosfomycin, MET metronidazole, CLA clarithromycin, CIP ciprofloxacin, AM amoxicillin, DO doxycycline, RIF rifampicin, M∆GI Membrane ∆G Insert. Increasing the negative value of membrane ∆G insert means increasing the antibiotic permeability

In Silico prediction of the membrane permeability of the seven tested antibiotics passage through *H. pylori* membranes was evaluated and the results indicated that FOS had greater membrane permeability compared to other tested antibiotics, with ∆G insert a value of − 37.537630 kcal/mol, followed by AM and DO, with ∆G insert values of − 31.255440 and − 29.848466 kcal/mol, respectively. Whereas, RIF and CIP showed moderate membrane permeability, with ∆G insert values of − 17.08 and − 13.84 kcal/mol, respectively. The lower membrane permeability was observed with MET and CLA, with ∆G insert values of − 6.04 and − 5.67 kcal/mol (Fig. 7 and Table 4).

**Table 4:** Computational exploration of the membrane permeability of *H. pylori* for seven different antibiotics

Ligands	Membrane permeability prediction				
	Membrane ∆G^*^ Insert^1^ (kcal/mol)	Membrane HDLD^2^ (kcal/mol)	Membrane GB^3^ (kcal/mol)	Membrane State Penalty^4^(kcal/mol)	Log Perm RRCK^5^ (cm/s)
FOS	− 37.54	− 29.87	− 6.15	− 7.67	− 6.34
AM	− 31.26	− 24.45	− 10.30	− 6.81	− 6.39
DO	− 29.85	− 21.86	− 9.54	− 7.99	− 6.58
RIF	− 17.08	− 15.11	− 7.06	− 1.97	− 6.21
CIP	− 13.84	− 10.33	− 4.40	− 3.50	− 5.33
MET	− 6.04	− 6.04	− 5.02	0.00	− 4.38
CLA	− 5.67	− 3.61	− 6.09	− 2.07	− 5.68

1 Membrane ∆G Insert: the total free energy penalty for the ligand to change state and enter the membrane. This is the net of the energy of Membrane HDLD and Membrane State Penalty; 2 Membrane HDLD: the free energy penalty for the neutral form of the ligand in its conformation inside the membrane to enter the membrane (i.e., move from the high dielectric region to the low dielectric region, hence HDLD). 3 Membrane GB: an implicit membrane generalized born theory model closely reproduces the Poisson–Boltzmann (PB) electrostatic solvation energy profile across the membrane. 4 Membrane State Penalty: a tautomerization penalty is derived from possible tautomer states and their estimated relative populations. These two processes are combined as a state penalty, ∆G state, that represents the free energy cost for the permeant to adopt a particular neutral, tautomeric form for membrane permeation. RRCK Ralph Russ canine kidney cells: 5 Log Perm RRCK: logarithm of the RRCK permeability in cm/s. This property is optimized to reproduce RRCK permeability assay results, with fitted energy

*FOS* fosfomycin, *MET* metronidazole, *CLA* clarithromycin, *CIP* ciprofloxacin, *AM* amoxicillin, *DO* doxycycline, *RIF* rifampicin

*Partition energy “∆G” Insert prediction

The interactions of FOS combinations with different antibiotics against the six test strains are summarized in Table 5. The results indicated that all the examined combinations exhibited good synergistic activities FIC index < 1 and re-sensitized the test strains to the used antibiotics. Notably, 128 mg/l was the optimal concentration of FOS for synergetic interactions (FIC index < 1) with other antibiotics against HP-1 and HP-3, whereas 64 mg/l FOS was the optimal concentration for the same interactions against the other four test strains. Interestingly, MICs of CLA, AM, CIP and DO in double FOS combinations were decreased from the ranges (3.2–12.8 mg/l), (3.2–25.6 mg/l), (3.2–51.2 mg/l) and (3.2> 51.2 mg/l) to the ranges (0.0125–0.025 mg/l), (0.0125–0.05 mg/l), (0.025–0.05 mg/l) and (0.025–0.05 mg/l), respectively. Similarly, MICs of RIF and MET against the same strains were decreased from (1.6–3.2 mg/l) and (32–256 mg/l) to 0.05 and 8 mg/l, respectively. Remarkably, addition of MET to FOS combinations with CLA, AM, CIP, DO and RIF has increased the synergistic interactions by decreasing the MICs of these antibiotics to half the concentrations used in the double combinations.

**Table 5:** The fractional inhibitory concentrations of FOS combinations with six different antibiotics against six MDR *H. pylori* strains

Strains	Antibiotic combinations										
	Double						Triple				
	FOS/CLA	FOS/AM	FOS/DO	FOS/CIP	FOS/RIF	FOS/MET	FOS/CLA/MET	FOS/AM/MET	FOS/DO/MET	FOS/CIP/MET	FOS/RIF/MET
MIC mg/l (FIC index)											
HP-1	128/0.025 (0.50)	128/0.05 (0.50)	128/0.05 (0.50)	128/0.05 (0.50)	128/0.05 (0.52)	128/8 (0.53)	64/0.0125/4 (0.27)	64/0.025/4 (0.27)	64/0.025/4 (0.27)	64/0.025/4 (0.27)	64/0.025/4 (0.27)
HP-2	64/0.025 (0.50)	64/0.05 (0.50)	64/0.05 (0.50)	64/0.025 (0.50)	64/0.05 (0.53)	64/8 (0.53)	32/0.0125/4 (0.27)	32/0.0125/4 (0.27)	32/0.025/4 (0.27)	32/0.0125/4 (0.27)	32/0.025/4 (0.28)
HP-3	128/0.025 (0.50)	128/0.05 (0.52)	128/0.05 (0.50)	128/0.05 (0.52)	128/0.05 (0.52)	128/8 (0.63)	64/0.0125/4 (0.31)	64/0.025/4 (0.32)	64/0.025/4 (0.31)	64/0.025/4 (0.32)	64/0.025/4 (0.32)
HP-4	64/0.0125 (0.50)	64/0.0125 (0.50)	64/0.025 (0.50)	64/0.025 (0.50)	64/0.05 (0.52)	64/8 (0.56)	32/0.0063/4 (0.28)	32/0.0063/4 (0.28)	32/0.0125/4 (0.28)	32/0.0125/4 (0.28)	32/0.025/4 (0.29)
HP-5	64/0.0125 (0.50)	64/0.0125 (0.50)	64/0.025 (0.50)	64/0.025 (0.50)	64/0.05 (0.52)	64/8 (0.56)	32/0.0063/4 (0.28)	32/0.0063/4 (0.28)	32/0.0125/4 (0.28)	32/0.0125/4 (0.28)	32/0.025/4 (0.29)
HP-6	64/0.0125 (0.50)	64/0.025 (0.51)	64/0.05 (0.50)	64/0.05 (0.52)	64/0.05 (0.52)	64/8 (0.75)	32/0.0063/4 (0.38)	32/0.0063/4 (0.38)	32/0.025/4 (0.38)	32/0.025/4 (0.38)	32/0.025/4 (0.38)

*FIC* fractional inhibitory concentration, *MIC* minimum inhibitory concentration, *MDR* multidrug resistant, *FOS* fosfomycin, *MET* metronidazole, *CLA* clarithromycin, *CIP* ciprofloxacin, *AM* amoxicillin, *DO* doxycycline, *RIF* rifampicin, *FIC* index of combination

(A/B) = FIC _antibiotic A_ + FIC _antibiotic B_, FIC of antibiotic = MIC _antibiotic in combination_/MIC _antibiotic alone_, synergism (FIC index ≤ 1); indifference (1.0 < ∑FIC ≤ 4) and antagonism (∑FIC > 4)

**Fig. 8:**
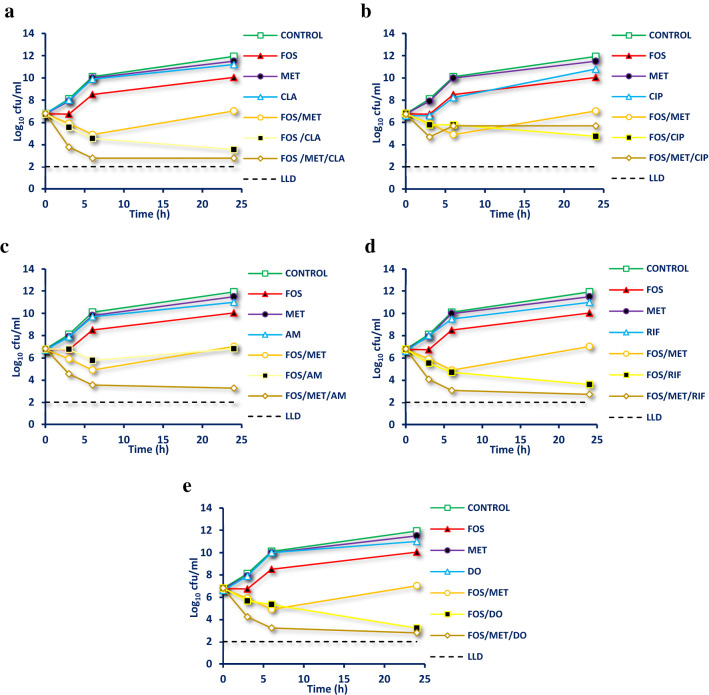
Time kill curves of FOS combined with five different antibiotics against HP-1 with presence and absence of MET. In single antibiotics and double combinations [FOS Fosfomycin 128 mg/l, MET metronidazole 8.0 mg/l, CLA clarithromycin 0.025 mg/l, AM amoxicillin 0.05 mg/l, DO doxycycline 0.05 mg/l, CIP ciprofloxacin 0.05 mg/l, RIF rifampicin 0.05 mg/l], In triple combinations [FOS Fosfomycin 64 mg/l, MET metronidazole 4.0 mg/l, CLA clarithromycin 0.0125 mg/l, AM amoxicillin 0.025 mg/l, DO doxycycline 0.025 mg/l, CIP ciprofloxacin 0.025 mg/l, RIF rifampicin 0.025 mg/l], LDD low limit of detection, cfu colony forming unit, **a** Time kill curve of FOS/CLA combination, **b** Time kill curve of FOS/CIP combination, **c** Time kill curve of FOS/AM combination, **d** Time kill curve of FOS/RIF combination, **e** Time kill curve of FOS/DO combination

Data on the time-kill kinetics of the tested single and combined antibiotics were consistent with those of the checkerboard experiments. The time-kill kinetics of antibiotics (CLA, MET, CIP, AM, RIF and DO) and their respective combinations with FOS against most resistant strain (HP-1) are presented in Fig. 8. As shown, kinetics of all single antibiotics against the representative strain were similar to those of the control, except for FOS, which caused an initial reduction in bacterial count within 3 h of post-treatment, followed by a considerable regrowth similar to the control after 6 h of treatment and lasted up to 24 h. Additionally, FOS combinations with AM, CIP and MET exhibited an initial reduction within 3–6 h post-treatment, followed by a considerable regrowth similar to the control after 24 h of treatment.

Figure 8 also shows that the combinations of FOS with CLA, DO and RIF produced bacteriostatic effects after 6 h of treatment, with 2.2, 2.1 and 2.08 log_10_ reduction in bacterial count, respectively. Moreover, these combinations exhibited bactericidal effects after 24 h post-treatment, with 3.2, 3.8 and 3.18 Log_10_ cfu/ml reduction in the initial inoculum, respectively. Notably, MET improved the activity of the combination of FOS with AM against HP-1 from a weak inhibition to a bacteriostatic effect within 3 h of treatment, with a reduction of 2.28 log_10_ cfu/ml, followed by bactericidal effects after 6 h of treatment and lasted up to 24 h, with a reduction of 3.28 and 3.56 log_10_ cfu/ml, respectively. Additionally, MET enhanced the bactericidal activity of FOS combinations with CLA, RIF and DO against the representative strain 24 h post-treatment, with the reduction in bacterial count increasing from 3.2, 3.18 and 3.6 to 4.04, 4.09 and 4.02 log_10_ cfu/ml, respectively. On the other hand, the presence of MET did not influence the activity of FOS/CIP combination against HP-1 (Fig. 2A–E). To the best of our knowledge, no previous study have investigated the bactericidal effects of these combinations against MDR *H. pylori* strains.

## Discussion

The effectiveness of standard therapeutic regimens for *H. pylori* infection has drastically reduced in recent years due to the increasing emergence of antibiotic resistance and the side effects of these regimens. Thus, new therapeutic options are urgently needed to combat the emergence of MDR *H. pylori* infections. Enhancing the efficacy of old antimicrobial agents represents one of the most feasible solutions for overcoming the high prevalence of MDR strains. In this study, we evaluate the synergistic potential of FOS combinations with a series of antibiotics used as first and second lines for the treatment of *H. pylori* infections. Additionally, the activity of these combinations against MDR *H. pylori* strains was also evaluated in the presence of MET.

Data in the present study showed that the MICs of CLA, MET, AM, CIP, RIF and DO against *H. pylori* strains were higher than the susceptible breakpoints listed in CLSI and EUCAST guidelines. The high resistance of *H. pylori* strains to the tested antibiotics can be explained by the excessive and uncontrolled consumption of antibiotics that are commonly used in the empirical treatment of *H. pylori* and other microbial infections such as respiratory and urinary infections as well as parasite infestation (Flores-Treviño et al. 2018; Savoldi et al. 2018; Choi et al. 2019)**.** Our findings are supported by data reported in previous studies, which suggested that the global consumption of fluoroquinolone and macrolide antibiotics were significantly increased by 64 and 19%, respectively, during the time period from 2000 to 2010 (Van Boeckel et al. 2014). Other studies mentioned that the failure of *H. pylori* eradication therapy is mainly due to the massive use of wasn’t for treating parasite infestations and uncontrolled consumption of macrolide and fluoroquinolones antibiotics in developing countries (Mégraud 2004; Kuo et al. 2017; Savoldi et al. 2018). Additionally, Klein and his co-worker reported that between 2000 and 2015, antibiotic consumption, expressed in defined daily doses, has increased 65% (Klein et al. 2018).

Fosfomycin is a bactericidal analog of phosphoenolpyruvate that has been previously been employed for uncomplicated urinary tract infections. The role of this antibiotic has been recently gained interest among physicians worldwide and the world health organization (WHO) defined it as critically important due to its potential efficacy against MDR Gram-positive and Gram-negative bacteria (Zdziebło et al. 2014; Falagas et al. 2016; Ruiz Ramos and Salavert Lletí 2019; Williams 2020). Additionally, many investigations mentioned that FOS may prove to be useful for *H. pylori* infection when the first-line antibiotic regimens fail (Barahona-Garrido et al. 2013; Boyanova et al. 2014; Falagas et al. 2016).

The docking results of the present study demonstrated that FOS had the highest binding affinity (docking score = − 5.310 kcal/mol) for *H. pylori* MurA, in comparison to the other tested antibiotics (CLA, MET, CIP, AM, RIF and DO), which used as the first and second lines for the treatment of *H. pylori* infections. Furthermore, FOS binds the protein active site of *H. pylori MurA* by forming 4 H bonds with Glu^190^, and 2H bond with Arg^236^ and Thr^307^. The obtained results are consistent with previous studies, which revealed that the bactericidal effects of FOS are due to its binding to the protein active site of the Mur A transferase, rendering it inactive. FOS inhibits the peptidoglycan biosynthesis by preventing the formation of UDP-N-acetylglucosamine-enolpyruvate from UDP-N-acetylglucosamine and phosphoenolpyruvate, resulting in the first step of bacterial cell wall synthesis is disrupted, which ultimately led to the destruction of the bacterial cell (Falagas et al. 2016; Díez-Aguilar and Cantón 2019).

Based on the State Penalty, the smaller value under alkaline and neutral conditions 0.6255, and 0.2534 kcal/mol, respectively showed better effect in comparison to low value under acidic condition (Greenwood et al. 2010; Madhavi Sastry et al. 2013). The docking score also affected by pKa value that depend on the pH value, where at pH value ranging from 8 to 11 the docking score increased and it was − 7.456 kcal/mol which mean high binding affinity as it forms 3 H-bonds with Arg^234^, and Thr^329^ and two salt bridges with Arg^234^ as it possess two negative charge, on the other hand at pH value of 7, the binding affinity was − 5.708 kcal/mol with the formation of 3 H-bonds with Arg^234^, and Thr^329^ and one salt bridges with Arg^234^ as it possess one negative charge, However at pH value range from 6 to 3, the docking score was 2.945 kcal/mol which mean very low binding affinity with the formation of 3 H-bonds with Arg^234^, and Asp^308^ under non ionized state.

Generally, most of antibiotics need to pass through at least one cellular membrane of Gram-negative bacteria to reach their intended target. Although tight binding of an antibiotic to its intended target is important for potency, poor membrane permeability often led to decrease the concertation of antibiotic inside the bacterial cell and reduce its efficacy (Wolak and Thorne 2013; Bennion et al. 2017; Domalaon et al. 2018)**.** Interestingly, our *In silco* data showed that FOS had the highest membrane permeability (membrane ∆G insert = − 37.54 kcal/mol) compared to other tested antibiotics, which exhibited low membrane permeabilities, with ∆G Insert ranging from − 5.67 to − 31.26 kcal/mol. From these findings, which agree with previous studies (Barahona-Garrido et al. 2013; Boyanova et al. 2014; Falagas et al. 2016), FOS could be a good suggestion as antimicrobial agent against MDR *H. pylori*, especially when the first-line antibiotic regimens fail.

In this study, the combinations of FOS with other tested antibiotics (CLA, MET, AM, CIP, RIF and DO) showed good synergistic effects (FIC index < 1) against all *H. pylori* strains and decreased the MICs of these antibiotics lower than the susceptible breakpoint. These findings obviously indicated that FOS might be adequate to re-sensitize the MDR *H. pylori* to these antibiotics in suitable combinations. The interaction between FOS and these antibiotics against MDR *H. pylori* was only investigated by one previous study, which supported our findings regarding the synergetic effects of FOS combinations with CLA, MET and AM against *H. pylori* strains (Blacky et al. 2005). Generally, our results are consistent with those reported by previous studies, which revealed that FOS/CIP combinations achieved synergistic effects against MDR strains of other Gram negative bacteria such as *Klebsiella pneumonia* (Yu et al. 2017), *Pseudomonas aeruginosa* (Walsh et al. 2016) and *E. coli* (Abu El-Wafa and Ibrahim 2020).

The data of time-kill curves of the single and combinations of used antibiotics were consistent with those of the checkerboard experiments. Time-kill curves of single antibiotics (FOS, CAL, MET, CIP, AM, RIF and DO) against representative strain (HP-1) showed a considerable regrowth similar to control after 24 h of post-treatment. Additionally, FOS combinations with AM, CIP and MET exhibited an initial reduction within 3–6 h post-treatment followed by a considerable regrowth similar to control after 24 h of post-treatment. These findings were in agreement with those mentioned by previous studies, which revealed that the regrowth phenomenon might be due to that the total bacterial burden contained two particular subpopulations with different susceptibility in which the selective amplification of resistant sub-population take over the preferential killing of the susceptible sub-population at a specified time of interaction (Tam et al., 2005; Sim et al., 2014).

Data in the present study showed that the combination of FOS with CLA, DO and RIF against HP-1 showed bacteriostatic and bactericidal effects after 6 and 24 h of post-treatment, respectively. Notably, MET enhanced the activity of FOS/AM combination against HP-1 from a weak inhibition to bacteriostatic effect within 3 h post-treatment, followed by bactericidal effects within 6 h post-treatment and lasted up to 24 h. Additionally, MET enhanced the bactericidal activities of FOS combinations with CLS, RIF and DO against the representative strain after 24 h of post-treatment, whereas the activity of FOS/CIP combination against HP-1 wasn’t affected in the presence of MET. To the best of our knowledge, no previous study investigated the bactericidal effects of these combinations against MDR *H. pylori* strains.

To date, only one study reported the synergistic interactions of FOS combinations with some of these antibiotics (CLA, MET and AM) against MDR *H. pylori* strains (Blacky et al. 2005). In general, the bactericidal activity of FOS/RIF combination was only reported against some MDR strains of Gram positive bacteria belonging to *Enterococcus faecalis*, *E. faecium* and methicillin-resistant *Staphylococcus aureus* (Simonetti et al. 2018). The combination of FOS and DO was also reported to exhibit synergistic and bactericidal effects against *Enterococcus faecium* (Davis et al. 2020).

## Conclusion

Based on in silico analysis, we found that FOS exhibited the highest predicted membrane permeability and binding affinity for *H. pylori* MurA*,* compared to other tested antibiotics, which used as the first and second lines for the treatment of *H. pylori* infections. Hence, FOS is potentially a promising antibiotic against *H. pylori* infection. Additionally, this antibiotic enhances the activity of CLA, DO, RIF and AM against MDR *H. pylori* by decreasing their MICs to the susceptible breakpoints. Moreover, the combinations of FOS with these antibiotics exert bactericidal effects against MDR *H. pylori,* especially with the presence of metronidazole. Thus, the combinations of FOS with CLA, DO, RIF and AM could be a promising option for treating MDR *H*. *pylori* infection, especially with the presence of metronidazole.
